# Integration of computer-simulated practical exercises into undergraduate medical pharmacology education at Mulungushi University, Zambia

**DOI:** 10.3352/jeehp.2020.17.8

**Published:** 2020-02-24

**Authors:** Christian Chinyere Ezeala

**Affiliations:** Department of Physiological Sciences and Medical Education Research Centre, School of Medicine and Health Sciences, Mulungushi University, Livingstone, Zambia; Hallym University, Korea

**Keywords:** Pharmacology, Computer simulation, Undergraduate medical education, Zambia

## Abstract

**Purpose:**

This study was conducted to determine whether a computer simulation of practical exercises in undergraduate medical pharmacology led to the realization of the intended learning outcomes.

**Methods:**

The study was a descriptive analysis of laboratory classes carried out using computer simulation programs. Five programs were used to teach practical pharmacology to undergraduate medical students at the Mulungushi University School of Medicine and Health Sciences. The study period was January 2018 to December 2019. The computer programs included a pharmacokinetics simulator (CyberPatient), organ bath simulator (OBSim), AutonomiCAL for simulating autonomic pharmacology, and Virtual Cat and Virtual Rat (RatCVS) for simulating cardiovascular pharmacology. Students utilized these programs during their pharmacology laboratory classes, wrote reports, and answered relevant clinical questions.

**Results:**

The 5 programs provided easy and precise platforms for students to explore concepts and demonstrate knowledge of pharmacokinetics, pharmacodynamics, autonomic and cardiovascular pharmacology, and their clinical applications.

**Conclusion:**

The programs were effective learning tools. Students’ learning was easily assessed based on their laboratory reports. Although the computer programs met medical students’ learning needs, wet laboratory exercises are also needed to meet the needs of students who require practical laboratory skills.

## Introduction

### Background/rationale

The pharmacology component of medical education programs is formulated with specific aims and objectives. Although these may vary from school to school, they generally emphasize understanding of the scientific basis of drug action and the pharmacological basis for their therapeutic uses. Laboratory classes complement lectures and provide hands-on exercises on the effects of drugs. However, there has been considerable debate on what constitutes effective pharmacology laboratory training in medical education. The time-honored approach comprised laboratory courses on experimental pharmacology and operation of a dispensing pharmacy. However, many scholars in medical education have argued that relevant pharmacology laboratory exercises should emphasize drug use in the clinical context [1]. This belief contrasts with the opinions of some teachers in the basic sciences and technical professions whose students require significant laboratory skills. No matter which side of the divide one favors, the importance of basic pharmacology laboratory exercises in reinforcing theoretical concepts should be emphasized.

A further issue in pharmacology is the need to reduce the use of laboratory animals in research and education. Animal rights groups oppose the use of animals for teaching and research. Furthermore, wet laboratory experiments are expensive because of the equipment and materials that are required. To address these issues, several software programs have been developed to simulate most basic pharmacology experiments with precision equaling or superseding wet lab experiments [2]. Reports have documented the successful use of these platforms for teaching and learning [3], and significant learning gains were reported by Ezeala et al. [4]. Formal integration of these programs into pharmacology teaching and learning appears attractive to medical schools with limited resources like those in sub-Saharan Africa. Therefore, the presented study investigated the use of this approach to complement the conventional medical curriculum of the Mulungushi University School of Medicine and Health Sciences, Zambia.

The School of Medicine and Health Sciences of Mulungushi University received accreditation from the Health Professions Council of Zambia and commenced operations in January 2018. Mulungushi University is a public university in Zambia, and its School of Medicine and Health Sciences is listed in the World Directory of Medical Schools. It operates a lecture-based 6-year curriculum, which includes 4 semesters of pharmacology, which extend from the third to the sixth semester. The modules covered within these 4 semesters include general pharmacology (including pharmacokinetics and pharmacodynamics), autonomic pharmacology, and pharmacology of the cardiovascular, respiratory, gastrointestinal, genito-renal, and endocrine systems. Other modules include neuropharmacology, chemotherapy, and clinical pharmacology and therapeutics.

### Objectives

This paper presents the application of computer simulations with freely downloadable software in the delivery of pharmacology laboratory classes. The aims of this study were to show that: the CyberPatient software is a useful tool for demonstrating pharmacokinetics laboratory exercises; the OBSim software could demonstrate pharmacodynamics concepts such as dose-response relationships, agonism, and antagonism; the AutonomiCAL software could be used to demonstrate the effects of the autonomic nervous system on eye function and to explore autonomic disorders of the eye; and that the Virtual Cat and Virtual Rat (RatCVS) programs are useful for demonstrating the effects of drugs on cardiovascular function.

In the author’s opinion, these 5 software programs could be successfully utilized to deliver the corresponding laboratory classes to medical students and achieve the intended learning outcomes.

## Methods

### Ethics statement

This study is a report of the effects of using certain software during the regular curriculum; therefore, no institutional review board approval was required, and there was no requirement to obtain informed consent from the students.

### Study design

The study is a descriptive analysis of the application of computer simulation software in pharmacology. The implementation of the exercises started with the beginning of the MBChB program in 2018.

### Content of the simulation exercises

Practical classes were customized to fit the topics in each module in the pharmacology courses. Computer simulations were used when possible in these practical classes. Five software programs were used in the study, including a pharmacokinetics program (CyberPatient) [5], an organ bath simulator, (OBSim) [6], AutonomiCAL [7], Virtual Cat [6], and RatCVS [6]. Each software program was fully described in the students’ manual, followed by an exercise that each learner carried out (alone or with a group of other students). Upon completion of each exercise, the students wrote individual reports. The reports included a title, aim(s), principles, procedures, observations, and conclusions. The students also answered any accompanying questions on pharmacological principles and/or therapeutic applications. Below are summaries of the various simulation exercises utilized in the delivery of the pharmacology courses at Mulungushi University.

#### Pharmacokinetics

Pharmacokinetics deals with the time course of drug concentrations in different body compartments and how this is affected by the physiological processes of absorption, distribution, metabolism, and excretion. Students carried out practical exercises in groups using computer-generated drug concentration–time data. They also utilized the CyberPatient software to demonstrate compartmental models for oral and intravenous administration of drugs. The clinical implications of drug distribution in 1 or 2 compartments were then discussed by the students in their reports. The CyberPatient software was also used to study the effects of different absorption rate constants on absorption kinetics. The students copied generated data to the clipboard and used it to plot concentration/time graphs using GraphPad Prism (GraphPad Software, La Jolla, CA, USA). Questions based on the observations were answered.

#### Pharmacodynamics

Pharmacodynamics deals with the mechanisms of drug action and the quantitative relationship between the dose and the magnitude of response. Graded dose-response studies and the effects of agonists and antagonists on drug receptors are usually investigated. For this, students used organ bath software (OBSim, from University of Strathclyde) to compare the efficacy and potency of histamine and carbachol on isolated guinea pig ileum. They also explored the antagonistic effects of mepyramine on histamine-induced contraction and the effects of hyoscine on carbachol-induced contractions. The obtained dose-response data were then used to create semi-log plots using GraphPad (GraphPad Software). The therapeutic implications were discussed. OBSim was also used to characterize the pharmacological properties of unknown drugs (1, 2, A, B, C, and D) using guinea pig ileum. Students determined whether the drugs were agonists or antagonists. Antagonism to histamine and carbachol were explored using these unknown drugs.

#### Autonomic pharmacology

For autonomic pharmacology, laboratory exercises are used to demonstrate the effects of the autonomic nervous system and autonomic drugs on specific organs and systems, such as the eye and the cardiovascular system [8]. The autonomic system has specific effects on eye reflexes. The AutonomiCAL software developed by the University of Melbourne, Australia was used to demonstrate the effects of autonomic drugs on pupillary reflexes in 4 patients with eye defects caused by derangements in autonomic transmission. AutonomiCAL has 2 screens. The first was used to screen patients’ eye reflexes to blinking and light. The second screen was used to examine the patients’ responses to 2 drugs chosen from atropine, pilocarpine, physostigmine, phenylephrine, cocaine, and amphetamine. A ruler in the program was used to measure pupil diameter. Using screen 1, students formulated a hypothesis about each patient. They used screen 2 to test the drugs on the patients’ eyes. They discussed their findings in groups and individually wrote a report explaining their findings.

#### Cardiovascular pharmacology

Two programs were used to demonstrate the effects of drugs on cardiovascular function. The Virtual Cat and RatCVS software programs developed by the University of Strathclyde, Glasgow, UK offer different platforms for CVS experimentation in whole animal models. The virtual cat can be used to demonstrate the effects of 15 standard drugs and 17 unknown drugs on blood pressure and heart rate, skeletal muscle contractions, and nictitating membrane twitches. Students used this program to investigate the effects of agonists, antagonists, and vasodilators on cardiovascular function. RatCVS simulates pithed and normal rat preparations, which are suitable for demonstrating the effects of drugs on the cardiovascular system without the superimposing effects of central baroreceptor reflexes. Pithing refers to the destruction of the spinal cord and the severance of central innervation to the cardiovascular system. The software gave options of using pithed or normal rat and provided the option of using 22 standard drugs and 10 unknown drugs. Using these systems, tutors provided guidelines according to which the students made their inquiries and provided scientific explanations for the observations.

## Results

**Fig. 1.**
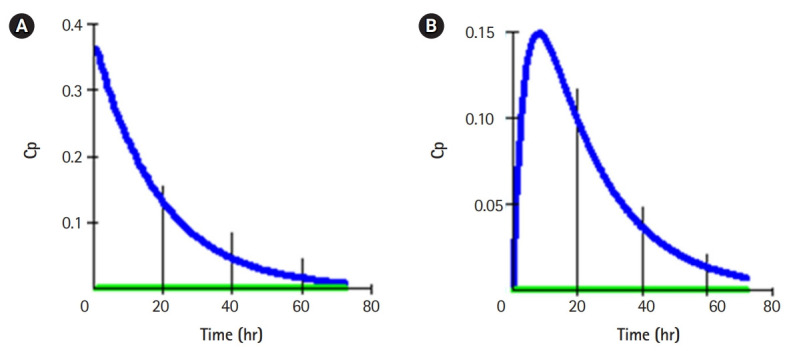
Drug distribution in a 1-compartment pharmacokinetic model following intravenous and oral administrations. Cp, plasma concentration.

**Fig. 2.**
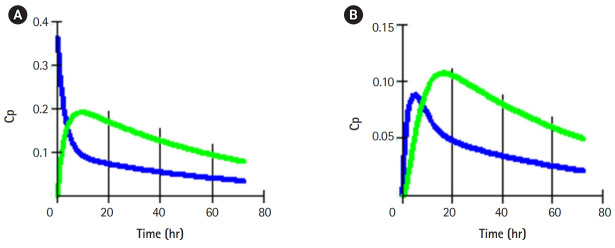
(A, B) Distribution into 2 compartments following intravenous and oral administrations, respectively. Cp, plasma concentration.

These simulation models were suitable for enhancing individual students’ inquiry and critical thinking skills relevant to the pharmacological basis of drug use. Students were able to demonstrate 1- and 2-compartmental models of drug distribution following single-bolus intravenous and oral administrations. Typical graphical plots of the models were shown below (Figs. 1, 2).

**Fig. 3.**
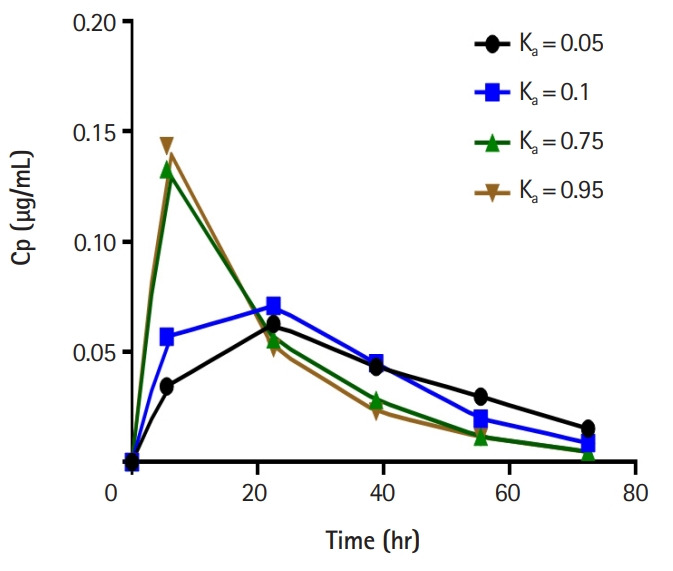
Effects of different absorption rate constants (Ka) on plasma drug concentrations. Cp, plasma concentration.

**Fig. 4.**
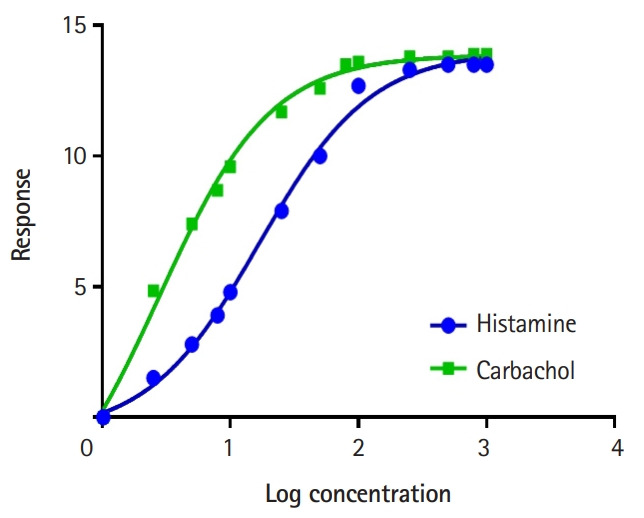
Comparison of the effects of histamine and carbachol on guinea pig ileum.

**Fig. 5.**
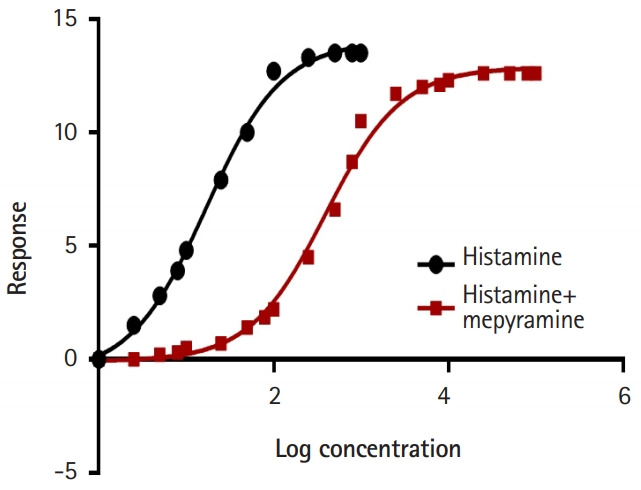
Antagonistic effect of mepyramine on histamine stimulation of guinea pig ileum.

**Fig. 6.**
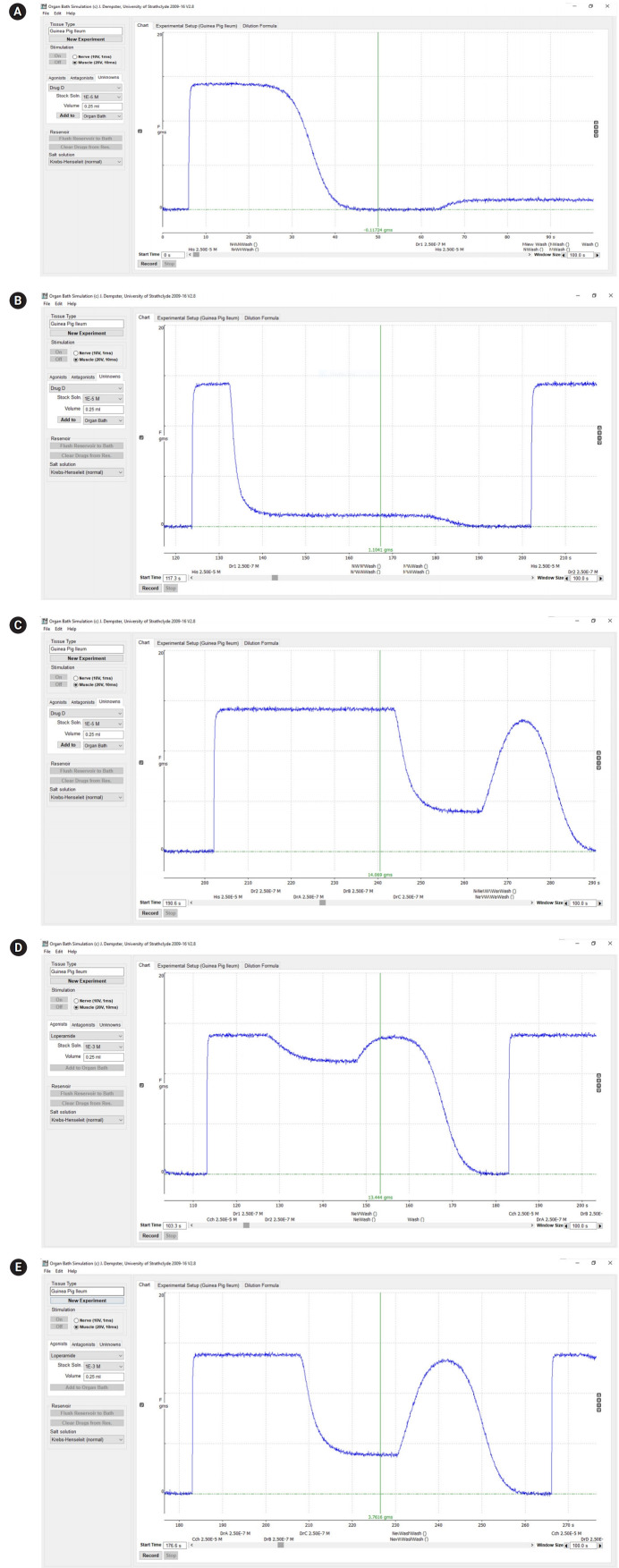
(A) Snippet from the organ bath simulator showing the agonistic effect of histamine and antagonism by drug 1. (B) Snippet from the organ bath simulator showing the agonistic effect of histamine, antagonism by drug 1, and no effect by drug 2. (C) Snippet from the organ bath simulator: drugs 2, A, and B have no effect on histamine-induced contraction, while drug C has an antagonistic effect. (D) Snippet from the organ bath simulator: drug 1 has no effect on carbachol-induced contractions, drug 2 has a weak antagonistic effect; and drug A has no effect. (E) Snippet from the organ bath simulator: drug B has no effect on carbachol-induced contractions, and drug C produces a remarkable antagonistic effect.

The students were also able to demonstrate the effects of differences in the absorption rate constant (Ka) on drug kinetics. Fig. 3 shows GraphPad plots of data from CyberPatient. For pharmacodynamics, the students successfully demonstrated graded dose-response relationships using OBSim. Figs. 4 and 5 show GraphPad plots of OBSim simulation data. The students were also able to characterize the pharmacological effects of standard and unknown drugs and to appreciate the concepts of agonism and antagonism. These effects are shown in the snippets presented in Fig. 6A–E.

## Discussion

### Key results

The purpose of this study was to evaluate the integration of computer simulations of pharmacology laboratory exercises into the medical education program at Mulungushi University. Our experiences with the 5 computer programs showed that this objective was successfully achieved and that the software provided learning experiences that met the needs of the pharmacology component of the curriculum.

### Comparison with previous studies

In classical pharmacokinetic laboratory exercises, drug concentrations are determined in body fluids such as plasma and urine, upon which basis students calculate relevant pharmacokinetic parameters such as the rate constant, area under the curve, bioavailability, clearance, elimination rate, and volume of distribution [9]. The effects of different drug formulations and routes of administration are commonly studied [10]. This study showed that the CyberPatient program was suitable for realizing most of these expectations and could be used to supplement wet laboratory exercises, which are more demanding. Classical methods for demonstrating dose-response relationships use an isolated organ/tissue bath with a kymograph or integrated with a data acquisition system such as ADInstrument’s PowerLab [11]. Simulation exercises with the OBSim software appeared to achieve the intended learning outcomes with good precision and convenience. This was also true of the AutonomiCAL program, which offered opportunities for students to study the effects of the autonomic system and autonomic drugs on the eye, and to appreciate the use of autonomic drugs in ophthalmic diagnoses.

The effects of drugs on the cardiovascular system could be demonstrated in several ways during laboratory classes. Isolated whole organ preparations such as the Langendorff perfusion system have been used to study the effects of drugs on the heart [12]. Organ baths could also be used to study the contractile or relaxant effects of drugs on the vasculature (e.g., using aortic rings) [13]. Nonetheless, the 2 computer programs used in this study proved to be suitable for realizing the intended learning outcomes of cardiovascular exercises.

### Limitations

These computer programs may suffice for the education of medical and dental students. However, for students in the basic sciences or technical educational programs whose learning objectives include the acquisition of laboratory skills, these programs may only serve as supplements to regular wet laboratory classes. A limitation of the study is that it did not report on students’ perceptions of the teaching approach. However, several articles have previously addressed this issue [14] and the learning gains achieved by using computer simulations [4].

### Conclusion

Although several articles have reported on the use of computer-aided learning in pharmacology education [14,15], this article provides a concise and comprehensive description of the application of simple free-to-download computer programs to simulate pharmacology laboratory exercises. The author believes that these programs could be an excellent resource for medical pharmacology teachers at institutions that do not have expensive laboratory equipment and consumables required for wet laboratory experiments and obviate the need to construct and maintain standard animal housing facilities. Students have the convenience of running the computer exercises at suitable times at the University’s Resource Center or at home using their personal laptops. This approach also exposed the students to the application of computer programs in medical education and data analysis using GraphPad.

## Supplementary materials

Supplement 1. Audio recording of the abstract.
